# The Prognostic Value of Non-Predominant Micropapillary Pattern in a Large Cohort of Resected Invasive Lung Adenocarcinoma Measuring ≤3 cm

**DOI:** 10.3389/fonc.2021.657506

**Published:** 2021-05-07

**Authors:** Hua Zhang, Wuhao Huang, Chang Liu, Giuseppe Giaccone, Xiaoliang Zhao, Xiaoyan Sun, Jingjing Li, Runfen Cheng, Qiujuan Huang, Huilan Mo, Zhenfa Zhang, Bin Zhang, Changli Wang

**Affiliations:** ^1^ Department of Lung Cancer, Tianjin Medical University Cancer Institute and Hospital, National Clinical Research Center for Cancer, Tianjin Lung Cancer Center, Key Laboratory of Cancer Prevention and Therapy, Tianjin’s Clinical Research Center for Cancer, Tianjin, China; ^2^ Sandra and Edward Meyer Cancer Center, New York, NY, United States; ^3^ Department of Lung Cancer Pathology, Tianjin Medical University Cancer Institute and Hospital, National Clinical Research Center for Cancer, Tianjin Lung Cancer Center, Key Laboratory of Cancer Prevention and Therapy, Tianjin’s Clinical Research Center for Cancer, Tianjin, China

**Keywords:** lung adenocarcinoma, micropapillary pattern, prognosis, survival, nomogram

## Abstract

The aim of this study was to analyze the influence of non-predominant micropapillary pattern in small sized invasive lung adenocarcinoma. A total of 986 lung adenocarcinoma patients with tumor size ≤3 cm were identified and classified according to the IALSC/ATS/ERS classification. Emphasis was placed on the impact of non-predominant micropapillary pattern on disease-free survival (DFS) and overall survival (OS). The relationship between lung adenocarcinoma subtype and lymph node involvement, EGFR mutation and KRAS mutation was also evaluated. A nomogram was developed to predict the probability of 3- and 5-year OS for these patients. The concordance index and calibration plot were used to validate this model. Among all 986 patients, the percentages of lymph node involvement were: 58.1, 50.0, 33.5, 21.4, 21.1, 10.9, 0, and 0% for micropapillary predominant, solid predominant, acinar predominant, papillary predominant, invasive mucinous adenocarcinoma (IMA), lepidic predominant, minimally invasive adenocarcinoma (MIA), adenocarcinoma *in situ* (AIS), respectively. The frequency of EGFR mutation in the cases of lepidic predominant, acinar predominant, MIA, micropapillary predominant, papillary predominant, solid predominant, IMA, and AIS were 51.1, 45.2, 44.4, 36.8, 29.3, 26.8, 8.3, and 0%, respectively. A non-predominant micropapillary pattern was observed in 344 (38.4%) invasive adenocarcinoma (IAC), and its presence predicted a poorer DFS (median: 56.0 months vs. 66.0 months, *P <*0.001) and OS (median: 61.0 months vs. 70.0 months, *P <*0.001). After propensity score matching, non-predominant micropapillary pattern retained its unfavorable effect on DFS (*P* = 0.007) and OS (*P* = 0.001). Multivariate analysis showed that non-predominant micropapillary pattern was identified as an independent prognostic factor for DFS (*P* = 0.003) and OS (*P <*0.001) in IAC. The nomogram showed good calibration and reliable discrimination ability (C-index = 0.775) to evaluated the 3- and 5-year OS. This retrospective analysis of patients with small sized IAC suggests the value of non-predominant micropapillary pattern to predict poor prognosis. A reliable nomogram model was constructed to provide personalized survival predictions.

## Introduction

Lung cancer remains the leading cause of malignancy-related death worldwide and in China (1). Adenocarcinoma is the most common histological subtype of lung cancer. Most lung adenocarcinomas are composed of mixed subtypes rather than comprising a single subtype (2). As a result of the existence of mixed histological subtypes, lung adenocarcinoma is characterized by a wide spectrum of radiological, clinical, pathological and molecular heterogeneity (3). In 2011, the International Association for the Study of Lung Cancer (IASLC), the American Thoracic Society (ATS) and the European Respiratory Society (ERS) released the new histological classification of lung adenocarcinoma (4). Meanwhile, this classification was adopted in the World Health Organization classification of lung tumors in 2015 (5). According to the new classification, invasive adenocarcinoma (IAC) includes five growth patterns: lepidic, acinar, papillary, micropapillary and solid.

Several studies have confirmed the prognostic value of the new lung adenocarcinoma classification (6–10). The five growth patterns were further grouped into three types, with different prognoses: lepidic (favorable), acinar and papillary (intermediate) and micropapillary and solid (poor). As a newly added subtype, micropapillary pattern has a unique pathological feature, that is, tumor cells grow in papillary tufts lacking fibrovascular cores that appear detached or connected with alveolar wall (9). Several studies have reported that patients with micropapillary-predominant adenocarcinoma have a poorer clinical outcome than those with the other subtypes (6–10). Recently Zhao et al. showed that patients with micropapillary pattern had a worse prognosis even if their pattern is not predominant (11). However, the prognostic value of non-predominant micropapillary pattern in lung adenocarcinoma has not been clearly described. Moreover, in most studies, adenocarcinoma *in situ* (AIS) (a non-invasive lesion) and invasive subtypes have been considered as part of one group, i.e. stage I. The 8th TNM classification sets AIS lesions apart from invasive lesions and categorizes AIS as stage 0 (12). Further studies are needed to confirm whether the presence of a non-predominant micropapillary pattern is an independent prognostic indicator in the 8th TNM classification.

With the application of high-resolution computed tomography (CT) and low-dose CT screening, more and more small sized lung tumors, especially lung adenocarcinoma, are identified. In fact, tumor size is a strong prognostic factor, which might affect adjuvant treatment (13). According to the 8th TNM classification of lung cancer, small-sized (≤3 cm) tumors are subdivided into ≤1.0 cm, >1 to ≤ 2 cm and >2 to ≤3 cm subgroups (12). Despite the overall good prognosis of patients with small-sized lung adenocarcinoma, there is heterogeneity in clinical outcome. We need better tools to identify patients who are at higher risk of recurrence and death.

In this study, we aimed to investigate the effect of non-predominant micropapillary pattern on disease-free survival (DFS) and overall survival (OS) in patients with IAC of small size (≤3 cm) in a large single center patient cohort who were radically resected. We also investigated the relationship between histological subtypes and lymph node involvement, EGFR mutation and KRAS mutation. Furthermore, we constructed a nomogram to predict the OS at 3- and 5-year.

## Materials and Methods

### Patients

Between January 2011 and January 2015, 1035 patients with tumor size ≤3 cm solitary lung adenocarcinoma underwent surgery in Tianjin Medical University Cancer Institute and Hospital. Of the 1,035 patients, 49 were excluded because of the following criteria: preoperative chemotherapy, radiotherapy, or other therapies (n = 17), the concomitant presence of other malignancies (n = 25), and positive surgical margins (n = 7). In total 986 patients were included in the current study. All patients were staged according to the 8th TNM Classification (12). This study was approved by the institutional review board of Tianjin Medical University Cancer Institute and Hospital. All informed consent was waived due to the retrospective nature of this study. Before analysis, all information of enrolled patients was anonymized and de‐identified.

### Histological Evaluation

Two pulmonary pathologists (Runfen Cheng and Qiujuan Huang, with 10 and 5 years of experience in pulmonary pathology, respectively), who were blinded to clinical information, independently evaluated the slides. The discordant cases were discussed until a consensus achieved. The average number of slides per patient reviewed was six (range: 4–12). According to the IASLC/ATS/ERS classification (four), each tumor was reclassified using comprehensive histologic subtyping and categorized into the following subtypes: AIS, minimally invasive adenocarcinoma (MIA), IAC, and variants of invasive adenocarcinoma. IAC could be lepidic predominant, acinar predominant, papillary predominant, micropapillary predominant or solid predominant. Predominant pattern was defined as the pattern with the greatest percentage. Non-predominant pattern indicates the subtype occupied no less than 5% but was not predominant. In addition, variants of invasive adenocarcinoma included invasive mucinous adenocarcinoma (IMA) and others.

### Gene Mutation Analysis

All lung adenocarcinoma tissues were obtained from surgery. Briefly, tumor DNA was extracted by using a QIAamp DNA FFPE tissue kit (Qiagen, Crawley, UK). According to the instruction of the manufacturer, EGFR tyrosine kinase exons 18, 19, 20, 21, and KRAS exon 2 were analyzed with an amplification refractory mutation system based on polymerase chain reaction using the ACCB Gene mutation Detection Kit (ACCB Biotech Ltd, Beijing, China).

### Data Collection and Follow Up

The following clinic-pathological variables were collected for each patient: age, sex, smoking status, tumor laterality, CEA, histological subtype, spread through air spaces, lymphovascular invasion, tumor size, lymph node metastasis, and pathological stage. Postoperative routine examinations, such as physical examination, chest CT scan, and neck and abdominal ultrasound were performed every three months for the first 2 years, and every 6 months thereafter for up to 5 years. After 5 years, follow-up frequency was once per year. Bone scan and brain magnetic resonance imaging or CT scan were done as clinically indicated.

### Construction and Evaluation of the Nomogram

In order to set up a quantitative method to predict the clinical outcome, we constructed a nomogram by integrating the independently clinical risk parameters identified in the multivariate Cox analysis. The concordance index (C-index) was calculated to evaluate the discrimination of the nomogram. It is generally recognized that the model has good discriminative ability when the C-index is greater than 0.70. Then, we draw the calibration plots to determine the performance of the nomogram. We used the bootstrapping method with 1,000 resamplings to implement the calibration plots. A 45° calibration curve represents an ideal prognosis prediction.

### Statistical Analysis

DFS was defined as the time from the date of the surgery until the first recurrence or the last follow-up. OS was defined as the time from the date of the surgery to the date of death or the last follow-up. To reduce the potential selection bias, propensity score matching (PSM) was used to adjust the confounding variables between the patients with non-predominant micropapillary pattern and those without micropapillary component. 1:1 ratio matching was performed, and matching covariates included tumor laterality, papillary subtype, solid subtype, spread through air spaces, CEA, tumor size, lymph node metastasis, and pathological stage.

The statistical analyses were performed by SPSS for Windows version 24.0 (SPSS, Inc., Chicago, IL). The correlation between clinic-pathological variables and histological parameter was analyzed by the chi‐squared test or Fisher’s exact test. The 5-year DFS rate and 5-year OS rate were calculated by the Kaplan–Meier method and compared using the log-rank test. The Cox proportional hazard model was used to calculate the hazard ratios (HR) and 95% confidence intervals (CI) for the DFS and OS in the univariate and multivariate analyses. *P*-values were two-sided, and values of <0.05 were considered significant. R software (version 3.6.3) was applied to constructed and evaluated the nomogram. The R package included rms and survival.

## Results

### Patient Characteristics

A total of 986 lung adenocarcinoma patients were enrolled in this retrospective study. The mean age was 60 years (range: 27–83 years), 423 were males (42.9%) and 563 females (57.1%). 567 patients (57.5%) had never smoked, 419 patients (42.5%) were current smokers or former smokers. The surgical approach employed in 430 patients (43.6%) was video-assisted thoracoscopic surgery (VATS) and it was open thoracotomy in 556 (56.4%) patients. Lobectomy was performed in 940 (95.3%) patients, wedge resection in 28 (2.9%) patients, segmentectomy in 11 (1.1%) patients, pneumonectomy in six (0.6%) patients, and sleeve lobectomy in one (0.1%) patient.

### Pathological Characteristics

The distribution of histopathological subtypes according to the IASLC/ATS/ERS classification is shown in **Supplementary Table 1**. The median tumor size was 2.0 cm (range: 0.3–3.0), including 12.8% (n = 126) with tumor size ≤1.0 cm, 43.9% (n = 433) with tumor size >1 to ≤2 cm, and 43.3% (n = 427) with tumor size >2 to ≤3 cm. Of 897 IAC, a non-predominant lepidic pattern was present in 274 tumors (30.5%). A non-predominant acinar pattern was observed in 342 tumors (38.1%), papillary in 140 tumors (15.6%), and solid in 120 tumors (13.4%). A non-predominant micropapillary pattern was identified in 344 tumors (38.4%).

### Relationship Between Histological Subtype and Lymph Node Involvement

Among all 986 patients, lymph node involvement was found 26.4% (n = 260) patients, including 56 (5.7%) N1 patients and 204 (20.7%) N2 patients. The percentages of lymph node involvement (N1 + N2) progressively decreased as follows: 58.1% (18 of 31), 50.0% (51 of 102), 33.5% (137 of 409), 21.4% (15 of 70), 21.1% (eight of 38), 10.9% (31 of 285), 0%, and 0% for micropapillary predominant, solid predominant, acinar predominant, papillary predominant, IMA, lepidic predominant, MIA, AIS, respectively.

### Relationship Between Histological Subtypes and EGFR Mutation and KRAS Mutation

Among all 986 patients, 615 received EGFR and KRAS mutation detection. EGFR mutation was found in 256 (41.6%) patients. Among the 256 EGFR mutations, 135 (52.7%) were L858R point mutation, 100 (39.1%) were deletions in exon 19, 10 (3.9%) were missense mutations of exon 18, six (2.3%) were insertions in exon 20, five (2.0%) were double mutations. The frequency of EGFR mutation in the cases of lepidic predominant, acinar predominant, MIA, micropapillary predominant, papillary predominant, solid predominant, IMA, and AIS was 51.1% (90 of 176), 45.2% (119 of 263), 44.4% (four of nine), 36.8% (seven of 19), 29.3% (12 of 41), 26.8% (22 of 82), 8.3% (two of 24), and 0% (0 of 1), respectively. The Kaplan–Meier curves for DFS and OS classified according to the EGFR status are shown in **Supplementary Figures 1A, B**. DFS and OS was significantly longer in patients with EGFR mutation compared with patients without EGFR mutation (*P* = 0.009 and *P* = 0.008, respectively).

KRAS mutation was found in 47 (7.6%) patients. The frequency of KRAS mutation in the cases of IMA, solid predominant, acinar predominant, papillary predominant, lepidic predominant, MIA, micropapillary predominant, and AIS was 29.2% (seven of 24), 13.4% (11 of 82), 8.4% (22 of 263), 4.9% (two of 41), 2.3% (four of 176), 1.1% (one of 9), 0% (zero of 19), and 0% (zero of one), respectively. The Kaplan–Meier curves for DFS and OS grouped by the KRAS status are shown in **Supplementary Figures 1C, D**. DFS and OS was significantly poorer in patients with KRAS mutation compared with patients without KRAS mutation (*P* = 0.037 and *P* = 0.016, respectively).

### Relationship Between Non-Predominant Micropapillary Pattern and Clinicopathological Variables

Of the 897 IAC cases, 31 (3.4%) were micropapillary predominant. 344 (38.4%) cases contained a non-predominant micropapillary pattern and 522 (58.2%) did not contain micropapillary pattern. We further analyzed the correlation between non-predominant micropapillary pattern and clinic-pathological variables in patients with non-micropapillary predominant IAC (**Table 1**). Our data showed that non-predominant micropapillary pattern was significantly correlated with tumor laterality (*P* = 0.003), papillary subtype (*P* = 0.006), solid subtype (*P* = 0.001), spread through air spaces (*P* = 0.003), CEA level (*P <*0.001), tumor size (*P* = 0.004), lymph node metastasis (*P <*0.001), and pathological stage (*P <*0.001).

**Table 1:** Relationship between non-predominant micropapillary pattern and clinic-pathological variables before and after PSM.

Variables	Before matching (n = 866)		*P* value	After matching (n = 460)		*P* value
	M+ not pren = 344	M−n = 522		M+ not pren = 230	M−n = 230	
Age						
≤60	173	283		116	127	
>60	171	239	0.258	114	103	0.304
Sex						
Male	151	225		105	87	
Female	193	297	0.818	125	143	0.089
Smoking status						
Yes	148	220		100	94	
No	196	302	0.798	130	136	0.571
Tumor laterality						
Left	148	173		84	90	
Right	196	349	0.003	146	140	0.564
Papillary subtype						
P−	276	385		183	172	
P+ not pre	37	98		33	41	
P pre	31	39	0.006	14	17	0.473
Solid subtype						
S−	261	339		187	180	
S+ not pre	38	67		28	29	
S pre	23	72	0.001	15	21	0.562
Spread through air spaces						
Yes	97	102		53	60	
No	247	420	0.003	177	170	0.448
Lymphovascular invasion						
Yes	55	86		39	28	
No	289	436	0.849	191	202	0.146
CEA (ug/L)						
≤5	223	406		171	177	
>5	114	105	<0.001	59	53	0.515
Tumor size (cm)						
≤1	23	64		16	17	
>1 to ≤2	148	244		99	93	
>2 to ≤3	173	214	0.004	115	120	0.850
Lymph node metastasis						
Yes	128	106		62	59	
No	216	416	<0.001	168	171	0.751
Pathological stage						
I	216	415		168	171	
II	22	29		13	10	
IIIA	106	78	<0.001	49	49	0.811

M−, micropapillary <5%; M+ not pre, micropapillary ≥5%, but not predominant; M pre, micropapillary predominant;

S−, solid <5%; S+ not pre, solid ≥5%, but not predominant; S pre, solid predominant;

P−, papillary <5%; P+ not pre, papillary ≥5%, but not predominant; P pre, papillary predominant;

PSM, propensity score matching.

### Survival Analysis of Predominant Patterns

Of the 986 patients, 73 lacked follow-up data; therefore, the remaining 913 patients were included in subsequent survival analysis. The median follow-up time for all the 913 patients was 68.0 months (range: 6–102 months). 277 (30.3%) patients developed recurrence and 201 (22.0%) patients died. The 5-year DFS rate and 5-year OS rate were 77.7 and 81.2%, respectively.

There was no recurrence or death in patients with AIS or MIA. The survival curves for the whole population are shown in **Supplementary Figure 2**. The 5-year DFS rates for the patients with the lepidic, acinar, papillary, solid, micropapillary predominant and variants of IAC were 91.1, 76.0, 75.9, 52.5, 37.9, and 60.3%, respectively. The 5-year OS rates were 92.5, 81.0, 78.8, 59.7, 40.8, and 64.0%, respectively. The survival analysis of histological subtypes in different tumor size groups is presented in **Supplementary Figure 3**.

### Survival Analysis of Non-Predominant Patterns

**Figure 1:**
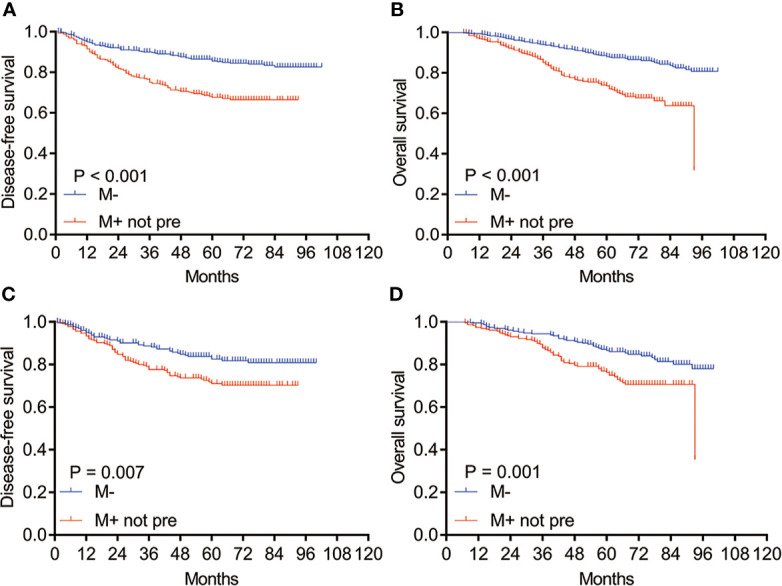
Kaplan–Meier survival curves of DFS and OS for patients with M− and M+ not pre before and after PSM. **(A)** DFS and **(B)** OS analysis for patients with M− and M+ not pre before PSM; **(C)** DFS and **(D)** OS analysis for patients with M− and M+ not pre after PSM. M−: without micropapillary pattern; M+ not pre: micropapillary pattern ≥5%, but not predominant; PSM, propensity score matching.

The presence of the non-predominant micropapillary pattern correlated with worse prognosis. The 5‐year DFS rate in the patients with non-predominant micropapillary component was significantly poorer than those without this component (67.6% vs. 85.5%, *P <*0.001; **Figure 1A**). The 5-year OS rate for patients with non-predominant micropapillary component was 73.5% in comparison to 88.0% for patients without this component (*P <*0.001; **Figure 1B**).

In order to more accurately assess the prognostic value of non-predominant micropapillary pattern, we performed the PSM on the basis of eight covariates, including tumor laterality, papillary subtype, solid subtype, spread through air spaces, CEA, tumor size, lymph node metastasis, and pathological stage. After PSM, 230 patients were included in each group, and no significant differences were found between two groups (**Table 1**). Consistent with the results before matching, we found that the DFS and OS in patients with a non-predominant micropapillary component were also significantly shorter than those without this component (*P* = 0.007 for DFS, **Figure 1C**; *P* = 0.001 for OS, **Figure 1D**).

**Figure 2:**
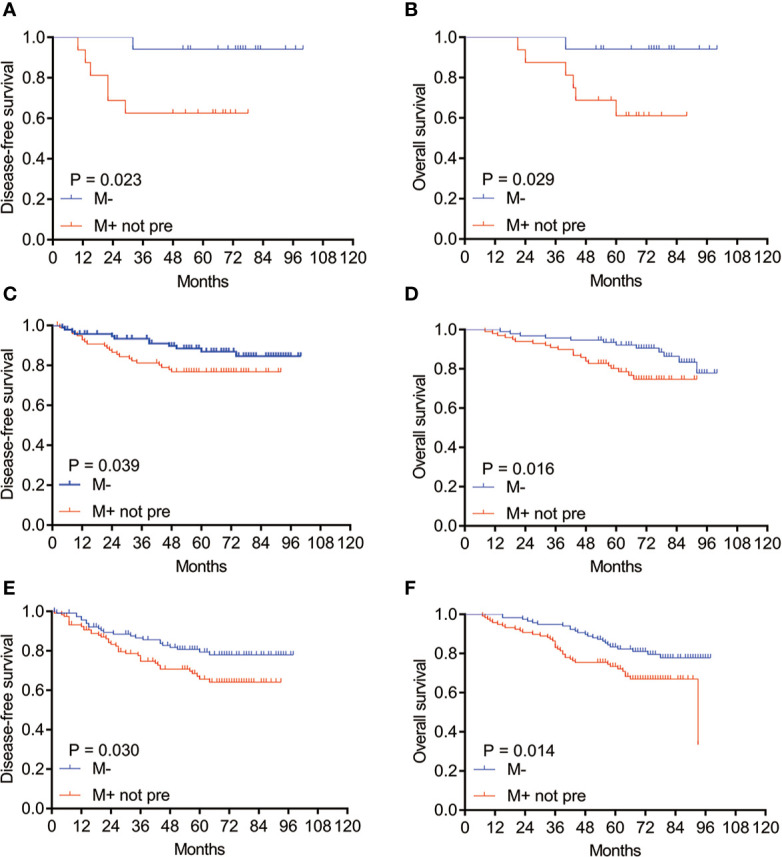
Kaplan–Meier survival curves of DFS and OS for patients with M− and M+ not pre subtypes in different tumor sizes after PSM. **(A)** DFS and **(B)** OS for patients with M− and M+ not pre subtype in tumor size ≤1 cm; **(C)** DFS and **(D)** OS in tumor size >1 cm, ≤2 cm; **(E)** DFS and **(F)** OS in tumor size >2 cm, ≤3 cm. M−: without micropapillary pattern; M+ not pre: micropapillary pattern ≥5%, but not predominant; PSM, propensity score matching.

**Figure 3:**
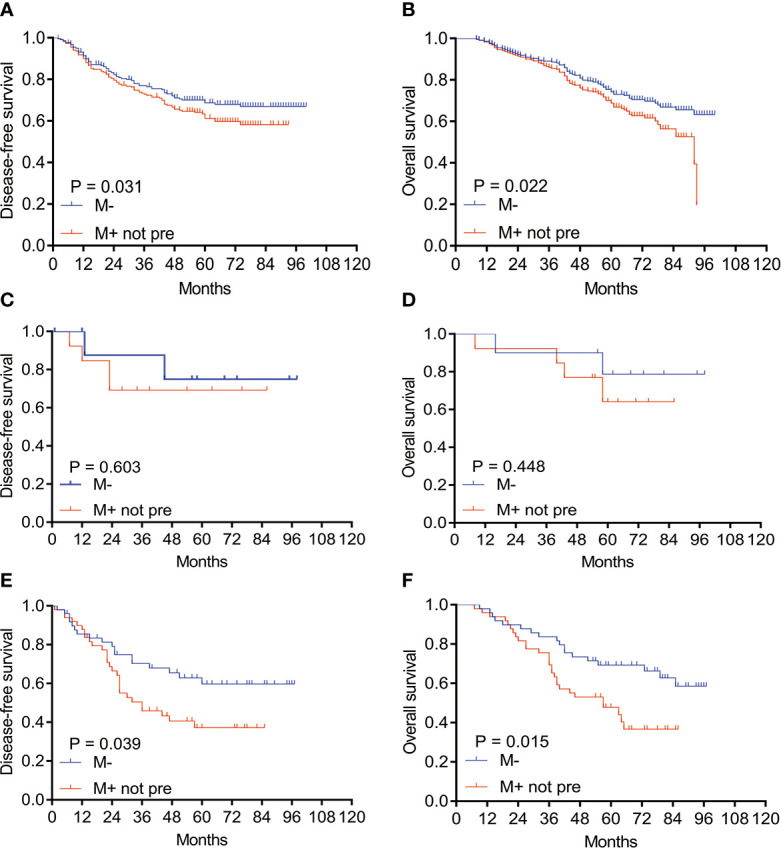
Kaplan–Meier survival curves of DFS and OS for patients with M− and M+ not pre subtypes in different pathological stages after PSM. **(A)** DFS and **(B)** OS for patients with M− and M+ not pre subtype in pathological I stage; **(C)** DFS and **(D)** OS in pathological II stage; **(E)** DFS and **(F)** OS in pathological III stage. M−: without micropapillary pattern; M+ not pre: micropapillary pattern ≥5%, but not predominant; PSM, propensity score matching.

After PSM, the survival analysis was performed in groups of different tumor size, our study showed that the survival of patients with non-predominant micropapillary pattern was also poorer than those without this component in the tumor size ≤1.0 cm, >1 to ≤2 cm, and >2 to ≤3 cm subgroups, respectively (**Figure 2**, all *P <*0.05). For different pathological stages, we found that the survival of patients with non-predominant micropapillary pattern was significantly shorter than those without this component in pathological stages I and III (**Figures 3A, B, E, F** for I and III stages, *P <*0.05). While we did not observe the significant difference between two groups in stage II patients (*P* = 0.603 for DFS, **Figure 3C**; *P* = 0.448 for OS, **Figure 3D**).

After PSM, the univariate analysis results showed that age (*P* = 0.029 and *P* = 0.022), smoking status (*P* = 0.004 and *P* = 0.009), CEA level (*P <*0.001 and *P <*0.001), solid subtype (*P <*0.001 and *P <*0.001), spread through air spaces (*P* = 0.017 and *P* = 0.034), pathological stage (*P <*0.001 and *P <*0.001), and non-predominant micropapillary pattern (*P* = 0.008 and *P* = 0.002) were significantly associated with DFS and OS, respectively (**Table 2**). Variables that were significant (*P <*0.05) in the univariate analysis were included in the multivariate analysis. After PSM, the multivariate analysis showed that the non-predominant micropapillary pattern remained an independent predictor of DFS (hazard ratio [HR] = 1.862, 95% confidence interval [CI] 1.233–2.810, *P* = 0.003) and OS (HR = 2.111, 95% CI 1.392–3.202, *P <*0.001) (**Table 3**).

**Table 2:** Univariate analysis of DFS and OS for non-micropapillary predominant IAC after PSM.

	DFS		OS	
	HR (95% CI)	*P* value	HR (95% CI)	*P* value
Age				
≤60	1		1	
>60	1.551 (1.045–2.301)	0.029	1.584 (1.067–2.350)	0.022
Sex				
Male	1		1	
Female	0.700 (0.474–1.034)	0.073	0.719 (0.486–1.062)	0.097
Smoking status				
No	1		1	
Yes	1.771 (1.197–2.620)	0.004	1.693 (1.144–2.507)	0.009
Tumor laterality				
Left	1		1	
Right	0.867 (0.583–1.289)	0.480	0.884 (0.594–1.316)	0.545
CEA				
≤5 ug/L	1		1	
>5 ug/L	2.207 (1.478–3.295)	<0.001	2.211 (1.480–3.301)	<0.001
Papillary		0.523		0.708
P−	1		1	
P+ not pre	1.331 (0.812–2.181)	0.257	1.233 (0.752–2.020)	0.407
P pre	1.108 (0.510–2.405)	0.796	1.062 (0.488–2.310)	0.879
Solid		<0.001		<0.001
S−	1		1	
S+ not pre	1.544 (0.881–2.704)	0.129	1.525 (0.870–2.671)	0.140
S pre	5.044 (3.053–8.335)	<0.001	4.177 (2.530–6.896)	<0.001
Spread through air spaces				
No	1		1	
Yes	1.658 (1.094–2.514)	0.017	1.567 (1.034–2.375)	0.034
Lymphovascular invasion				
No	1		1	
Yes	1.328 (0.798–2.211)	0.275	1.298 (0.780–2.161)	0.316
Pathological stage		<0.001		<0.001
I	1		1	
II	2.332 (0.998–5.449)	0.050	2.069 (0.886–4.833)	0.093
IIIA	4.350 (2.902–6.521)	<0.001	4.130 (2.759–6.182)	<0.001
Micropapillary				
M-	1		1	
M+ not pre	1.724 (1.154–2.574)	0.008	1.931 (1.285–2.902)	0.002

IAC, invasive adenocarcinoma; DFS, disease-free survival; OS, overall survival; HR, hazard ratio; CI, confidence interval; P pre, papillary predominant; P+ not pre: papillary ≥5%, but not predominant; P−, papillary <5%; S pre, solid predominant; S+ not pre, solid ≥5%, but not predominant; S−: solid <5%; M+ not pre, micropapillary ≥5%, but not predominant; M−, micropapillary <5%; PSM, propensity score matching.

**Table 3:** Multivariate analysis of DFS and OS for non-micropapillary predominant IAC after PSM.

	DFS		OS	
	HR (95% CI)	*P* value	HR (95% CI)	*P* value
Age				
≤60	1		1	
>60	1.243 (0.829–1.864)	0.292	1.357 (0.905–2.034)	0.139
Smoking status				
No	1		1	
Yes	1.436 (0.961–2.147)	0.077	1.349 (0.898–2.026)	0.150
CEA				
≤5 ug/L	1		1	
>5 ug/L	1.545 (1.005–2.376)	0.048	1.527 (1.098–2.427)	0.042
Solid		0.004		0.010
S−	1		1	
S+ not pre	1.112 (0.621–1.994)	0.721	1.211 (0.677–2.168)	0.519
S pre	2.623 (1.474–4.667)	0.001	2.433 (1.374–4.310)	0.002
Spread through air spaces				
No	1		1	
Yes	1.462 (0.924–2.312)	0.105	1.329 (0.846–2.088)	0.216
Pathological stage		<0.001		<0.001
I	1		1	
II	2.236 (0.949–5.267)	0.066	1.827 (0.777–4.298)	0.167
IIIA	3.357 (2.164–5.207)	<0.001	3.249 (2.107–5.010)	<0.001
Micropapillary				
M−	1		1	
M+ not pre	1.862 (1.233–2.810)	0.003	2.111 (1.392–3.202)	<0.001

IAC, invasive adenocarcinoma; DFS, disease-free survival; OS, overall survival; HR, hazard ratio; CI, confidence interval; S pre, solid predominant; S+ not pre, solid ≥5%, but not predominant; S−, solid <5%; M+ not pre, micropapillary ≥5%, but not predominant; M−, micropapillary <5%; PSM, propensity score matching.

Based on the different combinations of predominant growth patterns and non-predominant micropapillary pattern, IAC could be divided into four prognostic groups. Group 1 included lepidic predominant tumors without micropapillary pattern (L pre M−). Group 2 included lepidic predominant tumors with non-predominant micropapillary pattern (L pre M+) and acinar or papillary predominant tumors without micropapillary pattern (A/P pre M−). Group 3 included acinar or papillary predominant tumors with non-predominant micropapillary pattern (A/P pre M+) and solid predominant tumors without micropapillary pattern (S pre M−). Group 4 consisted of solid predominant tumors with non-predominant micropapillary pattern (S pre M+) and micropapillary predominant tumors (M pre). The 5-year DFS rates for these groups were 95.1, 85.0, 63.8 and 34.4%, respectively (*P <*0.001; **Supplementary Figure 4A**), and the 5-OS rates were 96.4, 87.9, 70.0, and 46.0%, respectively (*P <*0.001; **Supplementary Figure 4B**).There was no significant prognostic value of non-predominant lepidic, acinar, papillary, and solid patterns in IAC (**Supplementary Figure 5**).

### Establishment and Validation of the Nomogram

**Figure 4:**
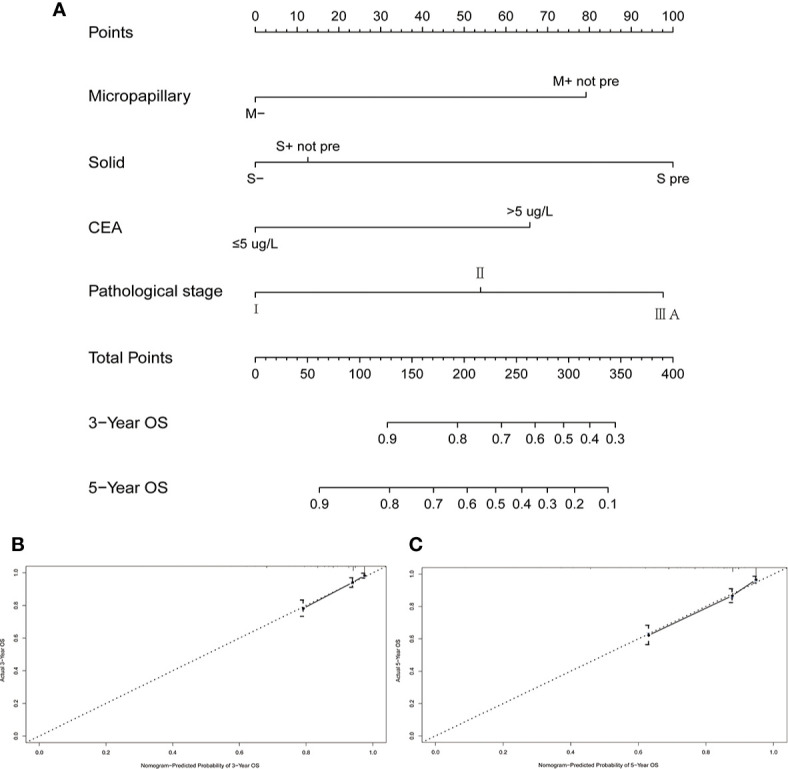
A nomogram model and its calibration plots for validation. **(A)** A nomogram to predict the 3- and 5-year OS rates for patients with resected invasive lung adenocarcinoma (≤3 cm); **(B)** calibration curve for predicting the 3-year OS; **(C)** calibration curve for predicting the 5-year OS. M−: without micropapillary pattern; M+ not pre: micropapillary pattern ≥5%, but not predominant; S pre: solid predominant; S+ not pre: solid ≥5%, but not predominant; S−: solid <5%.

We constructed a prognostic nomogram according to the indicators, including micropapillary pattern, solid pattern, CEA, and pathological stage, which were derived from the multivariate Cox analysis. A total of nomogram score was obtained on the basis of the sum of individual score from all predictive indicators. By calculating the nomogram score, we could estimate the approximate survival rates at 3 and 5 years for patients with resected invasive lung adenocarcinoma (≤3 cm) (**Figure 4A**). The C-index was 0.775, which indicated that the nomogram model had a good discrimination ability for OS. The calibration plots revealed good consistency relative to the actually observed outcomes, demonstrating that there was stability to predict survival of lung adenocarcinoma patients for the nomogram (**Figures 4B, C**).

## Discussion

In the present study, we showed a significant correlation between DFS and OS and the predominant histological subtypes in a cohort of 913 patients with radically resected solitary lung adenocarcinoma ≤3cm. Moreover, we showed that patients with tumors with the presence of non-predominant micropapillary pattern had a worse clinical outcome, and this was confirmed by the multivariate analysis after PSM. Furthermore, lymph node involvement in micropapillary and solid predominant tumors was significantly higher. EGFR mutations were more common in lepidic predominant adenocarcinoma. KRAS mutations were detected more often in IMA and no KRAS mutations were observed in micropapillary predominant adenocarcinoma.

Since the introduction of the new lung adenocarcinoma classification in 2011, several studies investigated the prognostic value of this new classification and showed that solid- and micropapillary-predominant subtypes have a worse prognosis compared with other subtypes of IAC (6–9, 14). However, previous studies have focused primarily on the relationship between predominant histological patterns and prognosis. The evaluation of prognostic value of non-predominant micropapillary pattern in patients with IAC has not been clearly described. Based on clinicopathological characteristics including micropapillary content, we constructed a nomogram model which was verified to have good predictive performance though C-index (0.775) and the calibration plots.

Sánchez-Mora et al. enrolled 92 stage IA lung adenocarcinoma patients and determined that the 5-year OS of patients with micropapillary component (<5%) was 77.0%, which was significantly higher than those with micropapillary component (≥5%) (54.0%) (15). Nitadori et al. reported that the presence of an micropapillary component (≥5%) was independently associated with the risk of recurrence in patients who underwent limited resection for small lung adenocarcinoma (≤2 cm) (16). However, in these studies, micropapillary predominant tumors was also included in the micropapillary component (≥5%) group, which may have some influence on the role of non-predominant micropapillary pattern in predicting the prognosis of lung adenocarcinoma. The results from a study by Tsubokawa et al. showed that the prognosis tended to be poorer for patients with acinar- and papillary-predominant patterns of micropapillary component ≥5% than micropapillary component <5% tumors (17). By analyzing the survival of 86 patients with acinar- and papillary-predominant subtypes, Matsuoka et al. found that a micropapillary and/or solid component ≥1% was associated with a worse clinical outcome (18). In a recent study by Zhao et al., patients with minor micropapillary component (>5%, but not predominant) had a shorter recurrence-free survival (RFS) and OS (11). However, these studies were published before the application of the 8th TNM classification. Whether the prognostic significance of non-micropapillary pattern is applicable to the 8th TNM classification needs further study.

Consistent with the previous results, we found that patients with AIS and MIA had a 100.0% 5-year DFS and OS, followed by lepidic-predominant patients. Patients with acinar- and papillary-predominant tumors shared an intermediate survival, while solid- and micropapillary-predominant tumors were associated with the worst prognosis. We next investigated the clinical significance of non-predominant patterns in IAC, especially for the non-predominant micropapillary pattern. Our data demonstrate that patients with a non-predominant micropapillary pattern have a poorer DFS and OS than those without this pattern, which is consistent with Zhao’s results (11). Furthermore, compared with those with micropapillary predominant, patients with non-micropapillary pattern showed a better DFS and OS. However, in Zhao’s study, there was no difference in RFS and OS between non-predominant micropapillary group and micropapillary predominant group. Our results are comparable to Tsubokawa et al.’s results, who examined 347 patients with clinical stage IA lung adenocarcinoma and reported that higher proportions (<5, 5–30 and ≥30%) of micropapillary patterns were associated with a poorer DFS (89.3, 76.0, and 48.1%, respectively; *P <*0.001) (17). Moreover, multivariate analysis showed that non-predominant micropapillary pattern was identified as an independent prognostic factor for DFS and OS after PSM.

On the other hand, we further performed subgroup analyses to investigate the prognostic value of non-predominant micropapillary pattern. Similar results were seen across different tumor sizes and pathological stages. Probably because of the small number of cases, a trend was found in pathological II stage. Meanwhile, we analyze the prognostic value of non-predominant lepidic, acinar, papillary, and solid pattern in IAC. We found these patterns did not possess statistically significant prognostic value. Mäkinen et al. reviewed 112 patients with surgically operated stages I–IV lung adenocarcinoma. A non-predominant lepidic component was observed in 24 tumors, and its presence predicted a better outcome (19). Cha et al. evaluated the clinical effect of the presence of solid subtype on the outcome in 511 lung adenocarcinoma patients with tumor size ≤3 cm and they found that the solid subtype (≥1%) had limited influence on the OS (20). Zhao et al. investigated 1,244 patients with lung adenocarcinoma and found that patients with a minor solid component (>5%, but not predominant) was a useful predictor of RFS and OS (11). The differences in the solid component between our cohort and Zhao et al.’s may be responsible for the difference in the RFS and OS. In addition, 39.2% patients had tumor size ≥3 cm in Zhao’s study. The significance of solid components in tumor size ≥3 cm may be greater than that in tumor size <3 cm. Therefore, future studies are needed to confirm the clinical significance of non-predominant lepidic, acinar, papillary, and solid patterns in IAC.

Another finding of our study is the lymph node involvement of the different histological subtypes. Yu et al. identified 2,268 operable lung adenocarcinoma patients with tumor size ≤3 cm and they found the percentages of lymph node involvement in solid-predominant and micropapillary was 47.6 and 47.2%, respectively, which is consistent with our results (21). However, no lymph node involvement was found in lepidic-predominant tumors in their study. In contrast, the percentages of lymph node involvement in lepidic-predominant was 10.9% in our study. In a recent study by Zhao et al, the lymph node involvement in lepidic-predominant tumor was 2.5% (11). Yeh et al. identified that the presence of micropapillary pattern is associated with occult lymph node metastasis (22). Among 30 lepidic-predominant tumors with lymph node involvement, 21 tumors contained micropapillary components, which may have an effect on lymph node involvement. Taken together, our results demonstrate that lung adenocarcinoma subtypes might have an effect on lymph node metastasis.

We also analyzed the relationship between histological subtypes and EGFR mutation and KRAS mutation. As the most common driver mutation in lung adenocarcinoma, EGFR mutation could predict response to the EGFR tyrosine kinase inhibitors (TKIs) (23). Thus, after the publication of the new classification of lung adenocarcinoma, several studies attempted to identify the relationship between EGFR mutation and histological subtypes. Even so, the relationship between them remains unclear. EGFR mutations were reported to be more frequent in lepidic (24), acinar (25), papillary (26) and micropapillary (27) predominant tumors. Recently, the results from a systematic literature review by Jiang et al. showed that EGFR mutations were more common in patients with lepidic predominant adenocarcinoma (28). Our results were consistent with this pooled-data analysis. It has been demonstrated that IMA (formerly mucinous bronchioloalveolar adenocarcinoma) correlates with the presence of KRAS mutations (29). In the present study, the frequency of KRAS mutation was highest in IMA, seven of 24 (29.2%). Tsuta et al. reported that KRAS mutations were most prevalent in IMAs (74.4%), followed by micropapillary predominant adenocarcinomas (16.2%) (26). However, in our study, no KRAS mutations were observed in micropapillary predominant subtypes. By analyzing 230 lung adenocarcinoma patients, Li et al. showed that the frequency of KRAS mutation in 13 micropapillary predominant tumors was also 0% (27). Ethnicity differences are known to contribute to different rates of EGFR and KRAS mutation in lung adenocarcinoma. In the previous studies, the prognostic value of EGFR mutation has not been consistent in resected cases. Some studies reported that patients with EGFR mutation had a better survival than those without EGFR mutation (30, 31), whereas others showed that EGFR mutation was not related with prognosis after surgery (32, 33). In Yoshizawa et al.’s study, the 5-year OS rate of 90 EGFR mutation patients was better than 77 patients without EGFR mutation (*P* = 0.015). However, the difference in DFS rates between these two groups did not reach significance (34). The differences in both DFS and OS were also not statistically significant between patients with KRAS mutation and those without KRAS mutation (34). In our large cohort study, EGFR mutation and KRAS mutation were prognostic indicators in a univariate analysis, but not in a multivariate analysis (data not shown). The disparity in the prognostic value of oncogenic mutations might be influenced by the different populations of enrolled patients, follow-up period or ethnicity. All patients were East Asian populations in our study, which is different from the TCGA database that contains mainly Caucasians. Our results indicate that the combination of histopathological analysis and molecular analysis might provide essential information for individualized treatment and prognostic stratification.

With the development of sequencing technology, several novel gene fusions were identified, which included neuregulin-1 (NRG1) fusions, MET fusions, and other fusions. These rare gene fusions were thought to be oncogenic drive mutations for non-small cell lung cancer (NSCLC), especially for lung adenocarcinoma (35). NRG1 fusion was very rare in NSCLC, and mainly occurred in invasive mucinous lung adenocarcinoma (36–38). Pan et al. reported that NRG1 fusions were identified in 0.36% lung adenocarcinoma patients (six of 1,681), and except for invasive mucinous adenocarcinoma, those fusions were also found in other subtypes, including solid, acinar and lepidic (39). MET fusions were relatively rare, and Plenker et al. found that those mutations were identified in 0.5% patients with lung adenocarcinoma (two of 337 cases) (40). Because of the relatively rare frequency of occurrence, the correlations between lung adenocarcinoma subtypes and these novel gene fusions are little reported, which were necessary to be investigated on the basis of large sample size in the future.

According to clinicopathological characteristics, including micropapillary pattern, solid pattern, CEA, and pathological stage, we constructed a prognostic nomogram which was proved to have good predictive performance by C-index and the calibration plots. Wang et al. developed a nomogram model based on clinical parameters, including solid and/or micropapillary components (predominant and minor patterns) in patients with pathological T1N0M0 invasive adenocarcinoma following lobectomy (14). In this study, Wang et al. grouped patients with solid and/or micropapillary into a group to build the model. The c-index of this nomogram was 0.703 for OS. In our nomogram model, the c-index was 0.775 for OS in patients with radically resected solitary lung adenocarcinoma ≤3 cm.

There are some limitations in our study. First, this is a retrospective and single-center study. Second, only lung adenocarcinoma patients with tumor size ≤3 cm were enrolled in this study. The prognostic value of non-predominant micropapillary pattern in patients with tumor >3 cm needs further study. Finally, our study lacked data of some data, such as ALK, ROS1, MET, NRG1 gene alterations, PD-L1 expression and so on, which were necessary to be further investigated in the future.

Despite these limitations, this study is informative. The prognostic value of new lung adenocarcinoma classification is not limited only to the predominant growth patterns. We want to highlight the important value of non-predominant micropapillary pattern in predicting prognosis for the patients with small sized IAC. Meanwhile, A reliable nomogram model was constructed to provide personalized survival predictions for patients with resected invasive lung adenocarcinoma (≤3 cm).

## Data Availability Statement

The datasets presented in this article are not readily available. The dataset about patient information may be used in other researches in the future. Requests to access the datasets should be directed to CW, wangchangli@tjmuch.com.

## Ethics Statement

The studies involving human participants were reviewed and approved by the institutional review board of Tianjin Medical University Cancer Institute and Hospital. Written informed consent for participation was not required for this study in accordance with the national legislation and the institutional requirements.

## Author Contributions

CW, BZ, and HZ conceived and designed the study. CL, XZ, XS, JL, RC, QH, and HM contributed to the data collection. GG and ZZ offered instructional suggestions. HZ and WH analyzed the data and participated in drafting the manuscript. CW, BZ, GG, and ZZ revised the article. All authors contributed to the article and approved the submitted version.

## Funding

This work was supported by National Key Research and Development Program of China (2017YFC1308704, 2016YFC0905501, 2016YFC0905500), National Natural Science Foundation of China (grant number 81772484, 81772488), Doctoral Start-up Fund of Tianjin Medical University Cancer Institute and Hospital (B1709 to HZ), and Tianjin Cancer Hospital Clinical Trial Project (C1705).

## Conflict of Interest

The authors declare that the research was conducted in the absence of any commercial or financial relationships that could be construed as a potential conflict of interest.
