# Supplementary figures and images for: The Prognostic Value of Non-Predominant Micropapillary Pattern in a Large Cohort of Resected Invasive Lung Adenocarcinoma Measuring ≤3 cm
Source: Front Oncol. 2021 May 7;11:657506. doi: 10.3389/fonc.2021.657506 (PMC8137894; doi:10.3389/fonc.2021.657506)

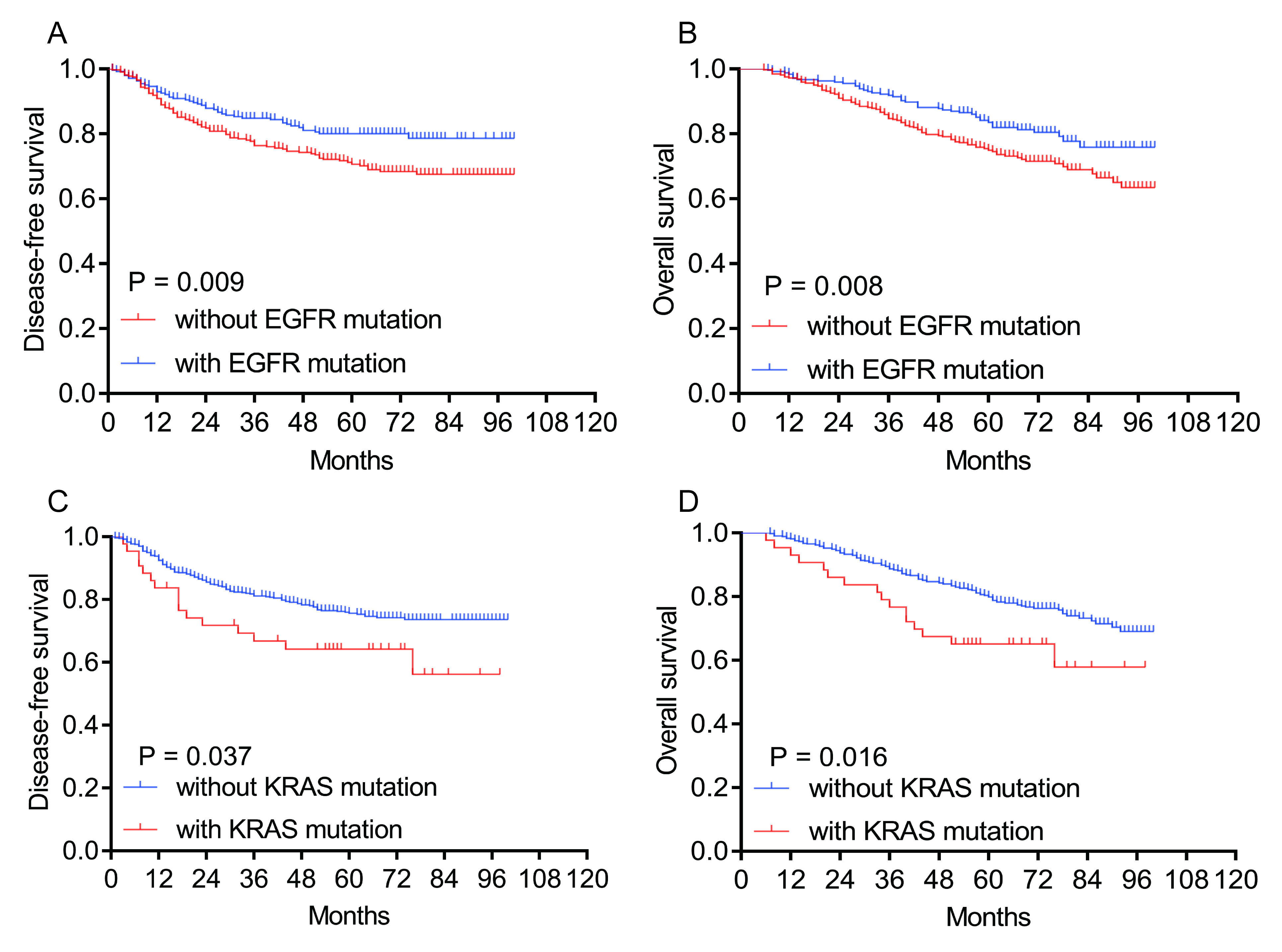

Supplement: Supplementary file 1 [file Image_1.tif]

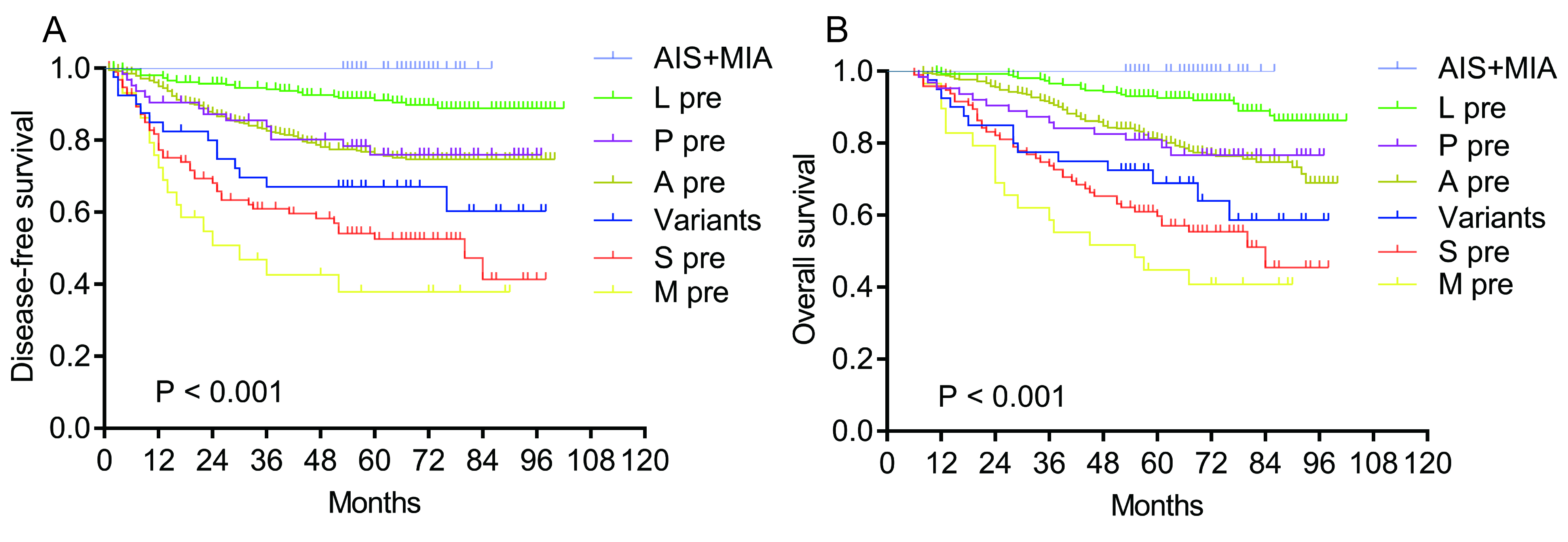

Supplement: Supplementary file 2 [file Image_2.tif]

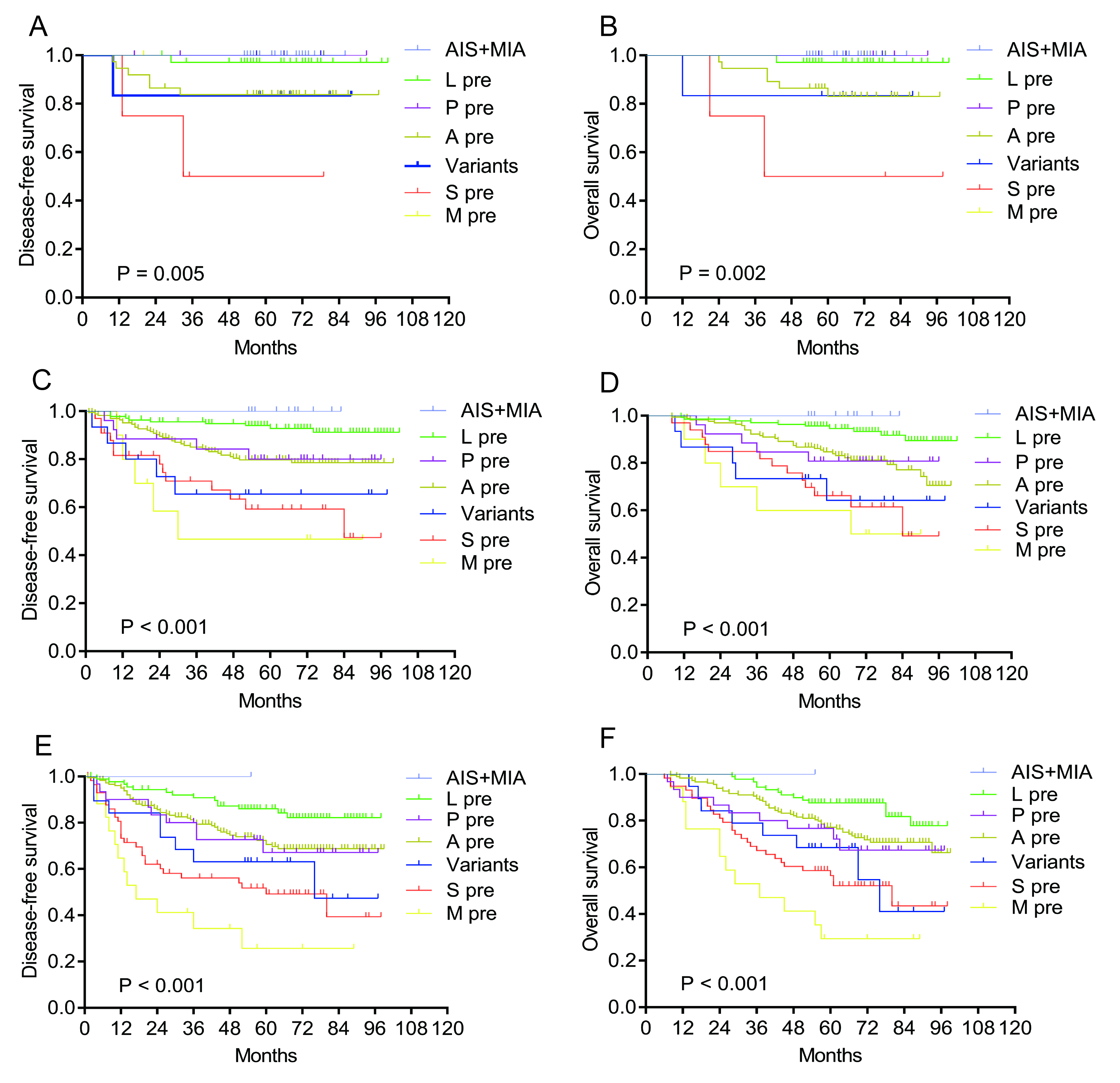

Supplement: Supplementary file 3 [file Image_3.tif]

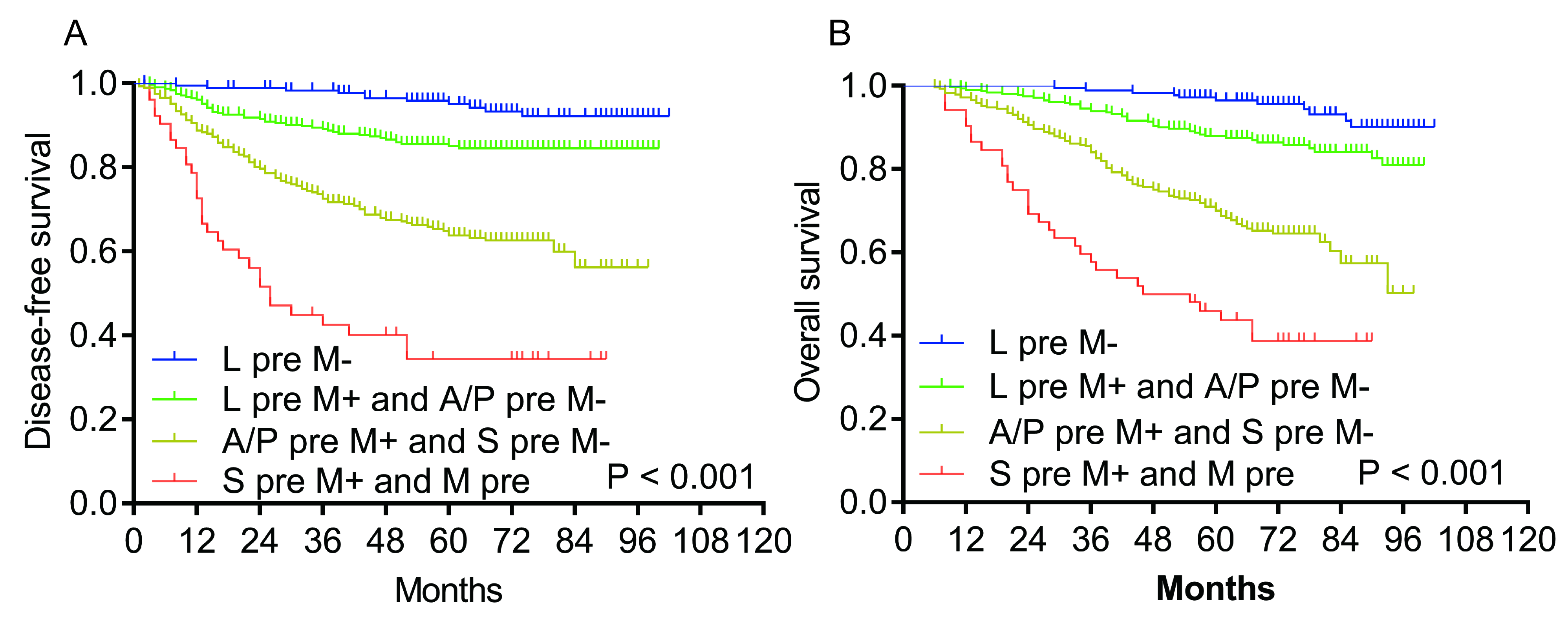

Supplement: Supplementary file 4 [file Image_4.tif]

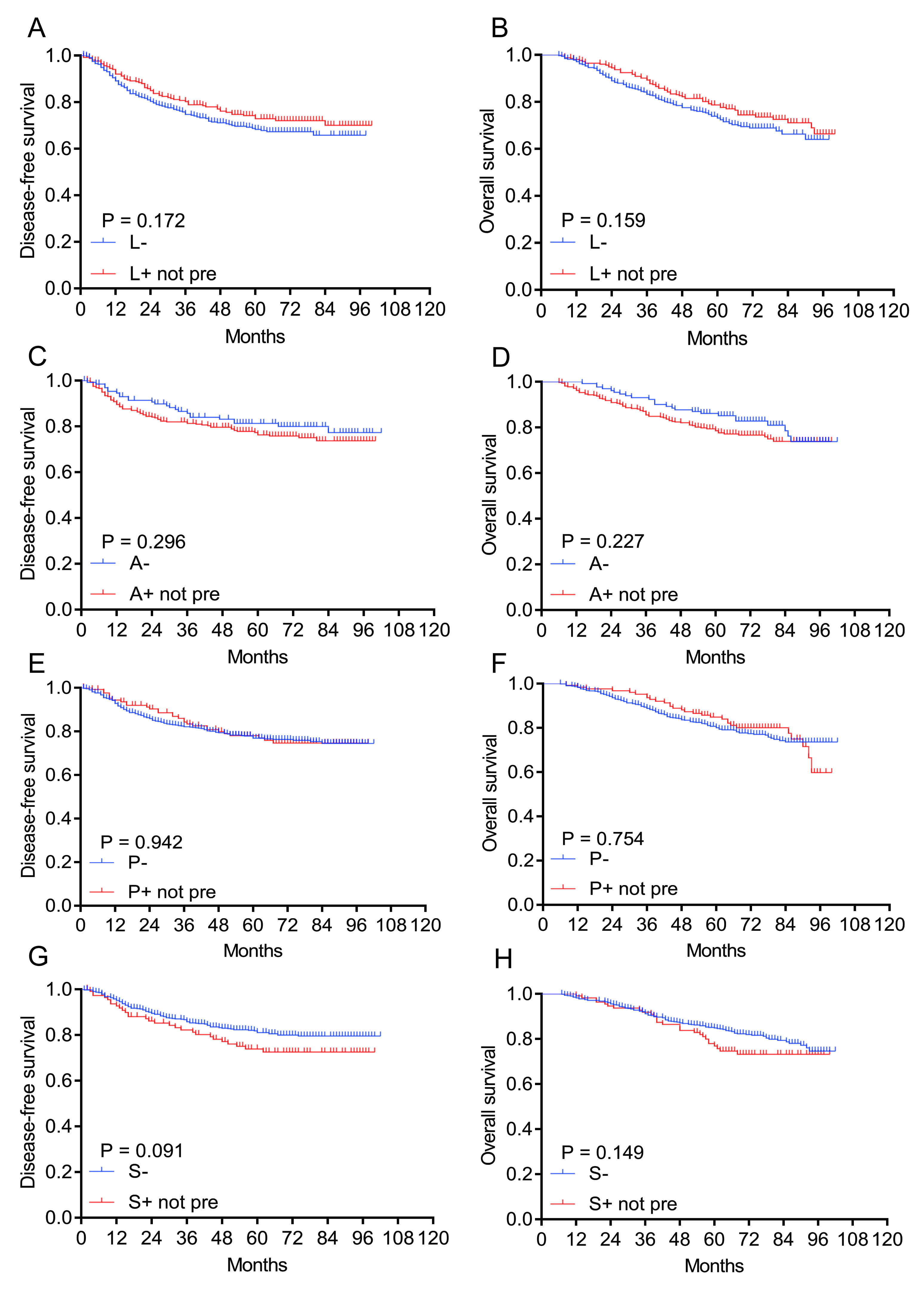

Supplement: Supplementary file 5 [file Image_5.tif]
