# Supplementary material for: The Prognostic Value of Non-Predominant Micropapillary Pattern in a Large Cohort of Resected Invasive Lung Adenocarcinoma Measuring ≤3 cm
Source: Front Oncol. 2021 May 7;11:657506. doi: 10.3389/fonc.2021.657506 (PMC8137894; doi:10.3389/fonc.2021.657506)
Supplement: Supplementary file 6 [file Table_1.docx]

**Supplementary Table 1.** Distribution of histological subtypes according to tumor size.

| Histological subtypes | All | Tumor size (cm) | | |
| --- | --- | --- | --- | --- |
|  |  | ≤1 | >1 to ≤2 | >2 to ≤3 |
| AIS | 2 | 1 | 1 | 0 |
| MIA | 44 | 30 | 14 | 0 |
| IAC |  |  |  |  |
| Lepidic predominant | 285 | 35 | 157 | 93 |
| Acinar predominant | 409 | 40 | 173 | 196 |
| Papillary predominant | 70 | 7 | 28 | 35 |
| Solid predominant | 102 | 5 | 34 | 63 |
| Micropapillary predominant | 31 | 2 | 11 | 18 |
| Variants of invasive adenocarcinoma |  |  |  |  |
| IMA | 38 | 6 | 12 | 20 |
| Colloid predominant | 4 | 0 | 2 | 2 |
| Enteric | 1 | 0 | 1 | 0 |
| Total | 986 | 126 | 433 | 427 |

AIS: Adenocarcinoma is situ; MIA: Minimally invasive adenocarcinoma; IAC: invasive adenocarcinoma; IMA: invasive mucinous adenocarcinoma.
